# Generative AI: A Disruptor to Health Professions Learner Assessment

**DOI:** 10.7759/cureus.96034

**Published:** 2025-11-03

**Authors:** Leo Morjaria, Bhavya Gandhi, Levi Burns, Matthew Sibbald

**Affiliations:** 1 School of Medicine, McMaster University, Hamilton, CAN; 2 Internal Medicine, McMaster University, Hamilton, CAN; 3 Radiation Oncology, University of Toronto, Toronto, CAN; 4 Cardiology, McMaster University, Hamilton, CAN

**Keywords:** artificial intelligence in assessment, generative artificial intelligence, health professions education, learner assessment, medical education

## Abstract

Since the widespread release of generative artificial intelligence (GenAI) tools in recent years, there has been a dramatic impact on health professions education, particularly in the context of learner assessment. As GenAI continues to evolve, traditional paradigms in health professions education are changing, requiring students, administrators, and educators to navigate ongoing disruption to their practice. We examine how GenAI will specifically impact assessment practices, offering three key postulates to guide future teaching and learning: (1) educators must re-examine assessment constructs to align with GenAI-enhanced learning environments, (2) GenAI can outperform median learner performance with appropriate prompting, and (3) GenAI will become a required tool for health professions assessment. Ultimately, we believe that rather than viewing the advent of GenAI as a threat, educators should harness its potential to empower students and augment learning.

## Editorial

Background

Artificial intelligence (AI) has driven significant societal change in a relatively short period of time. Generative AI (GenAI) is one of the primary drivers of this change, especially given the ability of large language model (LLM) platforms such as ChatGPT to communicate conversationally with users on virtually any topic. In education, GenAI is already having a large impact, but the level of familiarity among educators varies widely. Some educational leaders are early adopters, experimenting and implementing GenAI-powered tools, while others have taken a more skeptical position. In the current era of GenAI, assessors in health professions education are experiencing what may be akin to the stages of grief, with various levels of denial, anger, bargaining, depression, and acceptance. While others have recently applied the Kubler-Ross framework to describe the emotional transition of teachers in adapting to AI [1], our focus is narrower: we explore how these stages of grief manifest with regard to the impact of GenAI on health professions learner assessment. Many educators are grappling with the implications of GenAI for academic integrity, fairness, and traditional assessment methods in health professions education. As we move towards a future of synergy between assessors and GenAI, we put forward three postulates that will define the role of GenAI in this space. Instead of resisting change, our community stands to benefit from adaptation. This editorial presents a conceptual framework of three postulates to guide the responsible and human-centered integration of GenAI into health professions education.

More than just an emerging tool, GenAI is a mainstream technology that is reshaping entire industries. The technology underpinning these tools has been evolving for decades, but GenAI in its current form represents a new frontier in the ability to produce meaningful outputs in the form of text, images, and other media, in response to inputs, prompts, and feedback from non-expert users, unlike previous AI models. While many GenAI models are now available to the public, the LLMs developed by Facebook, OpenAI, and Google remain some of the most widely adopted. These LLMs are the core engines of modern dialogue-based GenAI tools, trained on massive datasets to understand and generate human-like text. From acting as a writing assistant and content creation tool to powering specialized medical applications to generate diagnostic insights, draft reports, or clinical documentation, GenAI has broad capabilities and transformative potential in health professions education [2]. A major challenge in integrating GenAI into health professions education is the low familiarity and training of educators and learners. This leads to a level of denial, insulating assessors and educators from fully embracing and adopting this new technology.

Denial

A 2022 web-based questionnaire exploring the familiarity of AI in medicine in five dimensions (demographics, concepts and definitions, training and education, implementation, and risks) provides insight into reasons for this denial [3]. This questionnaire was completed by 207 participants, of which 105 (50.7%) were medical doctors and 102 (49.3%) were medical students trained in all continents, with most from Europe, the Middle East, Asia, and North America. The results revealed no significant difference in the familiarity of AI between medical doctors and students (p=0.91), except that medical students perceived AI in medicine to lead to higher risks for patients and the field of medicine in general. Interestingly, another article published in 2024, two years after the public release of ChatGPT, found that 42% of medical education faculty had not used GenAI at the time of survey [4]. Even more surprisingly, only 6.1% of rehabilitation science learners reported routine use of these technologies [5].

Anger

As AI use cases continue to expand, many educational institutions do not have a standardized approach to managing this, leading to a reactionary approach. For example, Sciences Po, a leading university in France, banned ChatGPT to prevent plagiarism and fraud [6]. Furthermore, questions of whether AI poses an existential threat to higher education as a whole have been raised globally [7]. Specifically, tools such as ChatGPT have raised alarms on students using AI to complete assignments, potentially compromising academic integrity and the validity of traditional assessments [8]. Educators have also expressed fear that AI could diminish their evaluative authority and pedagogical significance, creating tension between innovation and tradition [9]. The concern has been likened to “AI doping,” where unchecked use of AI could undermine the very purpose of education, much like steroids once threatened the integrity of professional sports [10].

Concern exists regarding the capacity for GenAI to not only mimic human intelligence but also to surpass it in key areas. Stanford University reports that ChatGPT-4o exhibits behavioral and personality traits that are statistically indistinguishable from a random human, and in some areas including cooperation and task completion, it displayed superior performance [11]. Within undergraduate medical programs, ChatGPT 3.5, a now-outdated model, was shown to pass short-answer assessments at the pre-clinical level [12]. This has made it difficult for assessors to accurately and reliably identify text written by students or AI [13]. Beyond this, while plagiarism detectors have been able to keep assessments secure historically, AI detectors lack accuracy and have high rates of false positives and false negatives [14]. These findings highlight the need to redefine evaluations that assess student skills beyond deliverables that GenAI could reproduce, especially in health professions.

Bargaining

Given these new capabilities, many institutions are beginning to bargain with GenAI in an attempt to find a balance between its risks and rewards. Instead of outright banning GenAI, many organizations are creating guidelines, committees, and automation policies to mitigate risks while still leveraging GenAI’s potential. For example, the AI Advisory Committee at McMaster University in Canada represents a structured approach to AI integration, with experts across the institution coming together to define ethical and responsible AI policies [15]. Institutions are also experimenting with hybrid assessment models that combine AI-driven tools with human oversight to maintain academic rigor and fairness [2]. In line with this, student voices are being heard to identify concerns around GenAI use in higher education, including privacy and ethical issues, threats to practical skills development, and impact on career prospects. There is a growing demand for the creation of explicit ethical guidelines that clearly delineate acceptable GenAI use in academic settings [16].

Depression

As GenAI capabilities continue to accelerate, some educators are entering a stage of depression, marked by anxiety and uncertainty about their professional future. Many feel overwhelmed by the pace of GenAI advancements, raising fears about their ability to stay current with new technologies [17]. There are also growing concerns that traditional teaching roles are becoming less important as GenAI becomes increasingly integrated into personalized learning, case-based learning (e.g. virtual patients), and competency-based assessments [2].

Acceptance

With this shift in approach to GenAI, educators and assessors are moving towards acceptance, with an understanding that while GenAI is unlikely to entirely replace the modern health professions educator, those educators who do not adapt to this paradigm shift may lose relevance in favor of those who do. Part of this involves developing a synergistic approach to GenAI in education while recognizing that the capabilities of GenAI will evolve and may surpass human intelligence [18]. While GenAI is only beginning to assist human learners, planning for a future in which GenAI may support the expansion of medical knowledge is crucial to ensure harmony between health professions educators and GenAI.

As we accept the advantages of GenAI, we also need to accept the limitations. GenAI has intrinsic biases as it may be overtrained around a dominant view that may reinforce stereotypes [19]. Training can induce temporal, linguistic, demographic, cultural, political, and confirmation biases even when faced with contradictory evidence. The EU AI Act, provisionally agreed upon in December 2023, lays out requirements for training transparency and explainability [20]. Per the Act, explainability requires foundation model providers to account for relevant design choices in the AI system, including the quantity and suitability of datasets used for training and their possible biases. Further limitations include GenAI hallucinations and shallowness of responses in some settings, and learners and educators alike require an awareness of these shortcomings.

As assessors and institutions pass through these stages of grief, we propose three GenAI postulates for health professions assessors. First, health professions educators will need to carefully reconsider assessment constructs in a GenAI era. Clinicians are facing rising FDA approvals for AI technology [21], as well as extensive AI integrations [22, 23] in healthcare organizations, including through electronic medical record platforms [24]. Assessment constructs should recognize this and, while centering around core clinical competencies and activities, should mirror the environment in which future physicians will be practicing. Moreover, we recommend improved GenAI literacy of health professions educators, including improved awareness of GenAI tools and their evolving role in both education and routine patient care, and foundational training in how to access and use GenAI tools. Acknowledging that anyone can use GenAI without necessarily appreciating its weaknesses, we also prioritize critical thinking regarding GenAI technique and outputs for both educators and learners, including understanding the methods and sources used to create GenAI models, and unavoidable biases with these tools.

Second, GenAI should perform better than the median of a group of learners with appropriate prompting. As GenAI has evolved, research has shown us that the latest versions of leading GenAI models are able to surpass the median performance of human students on various standardized tests, including the LSAT, the Uniform Bar Examination, and the SATs [25]. The most recent and advanced GenAI models currently available are not only trained to reason and achieve PhD-level accuracy on benchmarks in physics, biology, and chemistry [26], but are also approaching the quality of work produced by industry experts across a broad range of professional tasks. In blind evaluations, OpenAI has found that models such as GPT-5 and Claude Opus 4.1 were frequently rated on par with, or superior to, expert-produced deliverables [27]. Interestingly, performance has more than doubled from GPT-4o (spring 2024) to GPT-5 (summer 2025) [27]. It was also noted that these models were able to complete these tasks roughly 100 times faster and 100 times cheaper than industry experts [27]. Of importance to the current discussion, GenAI performance also outperforms medical students in subject exams [28]. In fact, answers to consumer medical questions generated by a GenAI model, Med-PaLM 2, were preferred over physician answers by a panel of physicians [29]. Another recent tool is able to independently generate a differential diagnosis and provide superior assistance to human physicians compared to those equipped with standard search engines and medical resources [30]. Collectively, initial research with existing GenAI models across disciplines demonstrates outstanding GenAI performance in multiple domains, a trend that is only set to continue.

Third, GenAI will be a required tool for health professions assessment. GenAI is well-positioned to act as a content expert for the generation of assessments, including multiple-choice and short-answer questions [31]. In fact, the psychometric properties of AI-generated questions were shown to be the same as human-generated questions per a 2024 study [32]. Beyond assessment generation, GenAI may also assist in the grading of assessments. In the context of short-answer assessment in an undergraduate medical program, ChatGPT 3.5 was shown to be a viable assistant to human assessment with performance comparable to a single expert [33]. Together, this indicates that current GenAI models can provide meaningful support to both assessment generation and grading, with predicted improvement as newer models arrive. GenAI is similarly able to revolutionize case-based learning; a central component of most health professions curricula, learners stand to benefit from cases developed with GenAI and its ability to act as a virtual patient and provide customized, real-time feedback for students [34].

Conclusion

GenAI has changed the world and has started to change health professions. As experts attempt to grapple with this shift, health professions educators find themselves in the unique position to leverage this technology. Our three postulates provide a framework for recognizing this shift and easing the transition to a GenAI-enhanced era of teaching and learning. As GenAI becomes more integrated into assessment and education, its use must also remain responsible, ethical, and enhance rather than replace human judgment. While educators learn how to approach the disruption caused by GenAI to their profession, as the dust settles, it becomes evident that taking full advantage of GenAI technology is both possible and essential for the future of health professions education.
